# Diabetes, hypertension and mobility among Brazilian older adults: findings from the Brazilian National Household Sample Survey (1998, 2003 and 2008)

**DOI:** 10.1186/s12889-015-1956-2

**Published:** 2015-06-27

**Authors:** Clarissa de Matos Nascimento, Juliana Vaz de Melo Mambrini, Cesar Messias de Oliveira, Karla Cristina Giacomin, Sérgio Viana Peixoto

**Affiliations:** Rene Rachou Research Center, Oswaldo Cruz Foundation, Belo Horizonte, Minas Gerais Brazil; Research Department of Epidemiology and Public Health, University College London, London, UK; Secretaria Municipal de Saúde de Belo Horizonte, Belo Horizonte, Minas Gerais Brazil; Nursing School, Federal University of Minas Gerais, Belo Horizonte, Minas Gerais Brazil

**Keywords:** Hypertension, Diabetes, Mobility, Older adults, Item response theory

## Abstract

**Background:**

The rapid population ageing has been accompanied by a growing number of older adults experiencing chronic conditions, especially diabetes and hypertension, which are conditions associated to the decline in physical functioning. The aim of this study was to investigate changes in the strength of the association between mobility and two chronic conditions (hypertension and diabetes) in a large representative sample of Brazilian older adults over a ten year period.

**Methods:**

The data came from the Brazilian National Household Sample Survey (PNAD) of 1998, 2003 and 2008. The sample comprised 28,943 participants aged 60 years and older investigated in 1998, 35,042 in 2003 and 41,269 in 2008, totalling 105,254 older adults. The dependent variable was the physical mobility index (PMI) constructed based on the Item Response Theory (IRT) using five physical mobility indicators. The chronic conditions were self-reported and the confounders included: age, sex, schooling, ethnicity, family income, household composition, other co-morbidities and use of health services. The association between physical mobility (three different groups) and chronic conditions (hypertension and diabetes) was performed using multinomial logistic regression.

**Results:**

Over the ten year period the prevalence of hypertension increased from 44 % (1998), 49 % (2003) to 53 % (2008) (*p* < 0.001). Similar pattern was observed for the prevalence of diabetes: 10 % in 1998, 13 % in 2003 and 16 % in 2008 (*p* < 0.001). Overall, physical mobility showed a statistical significant association with both chronic diseases studied even after adjusting for potential confounders. The time-disease interaction term was significant (*p* < 0.05) for the two chronic conditions studied, and the strength of the associations decreased over the first five years, but it was not sustained between 2003 and 2008.

**Conclusions:**

Despite the increases observed in the prevalence of the hypertension and diabetes over the ten year period, the decrease in strength of the association with physical mobility during the first period could be explained by improvements in health services and treatment of older adults. Special attention should be given to the treatment and management of diabetes in order to avoid declines in physical mobility levels.

## Background

The preservation of the capacity to live independently and to function well during later life is important from both individual quality of life and public health perspectives. Mobility is a critical characteristic for functioning independently [1]. Those who lose mobility have higher rates of morbidity, hospitalization, disability, institutionalization and mortality. They also have a higher probability of developing depression and social isolation [2].

In addition to the basic activities of daily living (ADLs) and the instrumental activities of daily living (IADLs), physical functioning among older adults can be assessed by mobility level. The level of mobility can be measured by a self-reported scale starting with simple tasks like the ability to move from a bed to a chair to more physically challenging tasks such as short and long walks as well as climbing stairs [3]. Mobility is a very important indicator when investigating the relationship between physical functioning and sociodemographics, chronic conditions and health behaviours [4].

The Brazilian population is undergoing one of the fastest ageing processes worldwide. Ageing has consequently become more important for social policy development [5, 6]. In addition, there has been an increase in the proportion of older adults in Brazil presenting with multiple chronic disease, especially diabetes and hypertension, which are conditions associated to the decline in physical functioning [7, 8]. Hypertensive individuals have greater risk of developing physical functioning difficulties, dementia, depression and falls [7]. In addition, hypertension has an independent association with a decline in physical mobility [7, 9, 10]. Diabetes is a chronic disease with multiple causes [11] and recent studies have demonstrated that diabetic older adults show greater loss in mobility when compared to non-diabetic individuals [8, 12].

In Brazil, approximately half of the men and more than half of women aged 60 years and over reported previous diagnosis of hypertension, and the control of the disease (<140/90 mm Hg) is unsatisfactory [13]. The prevalence of diabetes also increase exponentially with age, from 0.5 % in those 18–24 years old to 21.4 % in those 65 years or older [14]. Primary care services have grown rapidly in Brazil since 1994, with the implementation of Family Health Program, leading to a reduction in rates of hospitalizations for many ambulatory care-sensitive chronic diseases, but this trend was not observed for hypertension (among male) and diabetes [15]. Considering this context, it is important to access the impact of these chronic conditions on the health of the elderly, especially with regard to physical functioning, which is directly linked to quality of life and successful ageing.

Therefore, the aim of this study was to investigate changes in the strength of the association between mobility and two chronic conditions (i.e. diabetes and hypertension) among Brazilian older adults between 1998 and 2008.

## Methods

### Study sample

The data came from the Brazilian National Household Sample Survey (PNAD) of 1998, 2003 and 2008. PNAD is a nationally representative survey conducted by the Brazilian Institute of Geography and Statistics (IBGE), that recruits participants using a three-stage complex probabilistic household sample and is representative of the national and regional levels. In the first stage municipalities are selected and divided in self-representative, with probability 100 % of belonging to the sample and non-representative which probability of belonging to the sample is proportional to the resident population. In the second stage, census tract are selected, which the probability of participation is proportional to the number of household in the tract. In the third stage, household are sampled in each census tract and the information relating to all household resident are collected through interview [16–18].

The sample comprised 28,943 participants aged 60 years and older investigated in 1998, 35,042 in 2003 and 41,269 in 2008, totalling 105,254 older adults eligible. The participants with information for all study variables considered for this analysis were 27,711 in 1998, 33,797 in 2003 and 39,500 in 2008. Thus, our final analytical sample comprised 101,008 older adults.

### Measures

#### Physical mobility

The dependent variable was the physical mobility index (PMI) based on Item Response Theory (IRT) [19] using the following mobility items: “Normally, because of a health problem, do you have difficulty in”: 1. Running, lifting weight, doing sports or doing heavy work?; 2. Pushing a table or doing a home repair?; 3. Going up a steep hill or stairs?; 4. Stooping or kneeling?; 5. Walking about 100 m? Possible response options to each item were: unable to do it; great difficulty; small difficulty and no difficulty at all. The internal consistency of the index based on the five physical mobility items mentioned above was tested using the Cronbach’s Alpha (α = 0.915). More detailed information regarding the IRT can be found elsewhere [19–21].

IRT scores were categorized into three groups due the asymmetric distribution of the score, with 27.2 % of the older adults without difficulty to perform the activities considered, and thus classified as group 1. The other two groups were defined based on the median score, excluding the individuals from the first group. As the value of the score represents the degree of difficulty in activities, the use of the median value could differentiate the group of respondents with some difficulty among those with moderate and great difficulty. Therefore, older adults were classified according to their level of difficulty to perform the five physical mobility items as follows: group 1 = no difficulty; group 2 = moderate difficulty and group 3 = great difficulty.

#### Diabetes and hypertension

Self-reported diagnosed diabetes and hypertension were the chronic diseases used in the analyses.

#### Covariates

We included age (60–69 years, 70–79 years and 80 and over), sex, years of schooling (none, between 1 and 3, 4 and over), ethnicity (white, non-white), and household composition (live alone, live with other people). The per capita family income was obtained from the income total household divided by the number of residents. Per capita family income were converted into Brazilian monthly minimum wages (1998 = US$ 110.17; 2003 = US$ 80.54 and 2008 = US$ 253.05) and grouped in tertiles. A variable called number of self-reported co-morbidities (none, 1 or more) was constructed based on the following question: “Have you ever been diagnosed by a medical doctor with the following health condition?” back problems, arthritis, cancer, diabetes, bronchitis or asthma, hypertension, heart disease, chronic kidney disease, depression, tuberculosis, tendinitis and cirrhosis. The use of health services was measured by both the number of medical consultations and hospitalization in the past 12 months. These potential confounders were chosen based in the literature [2, 9, 22, 23].

#### Statistical analyses

The physical mobility index was constructed using the Itm (latent trait model) package [24] from R software [25]. The other analyses were conducted using STATA version 13 (College Station, Texas, USA) for complex surveys design, considering the survey weights and the effect of sampling design [26]. The association between explanatory variables and survey years (1998, 2003 and 2008) were measure by Pearson’s chi-square test. Multinomial logistic regression was used to estimate the odds ratio, and their 95 % confidence intervals, of having physical mobility difficulties in relation to the presence of diabetes and hypertension, for each year. Group no difficulty was the reference category. The generalised Hosmer-Lemeshow test was used to assess the fitness of the models. The trends in the associations were evaluated by the year-disease interaction term in all models. The level of significance was 5 %.

## Results

Table 1 shows sample characteristics over the ten year period (i.e. 1998–2008). Overall, we found that there was a significant increase in the proportion of the oldest old group, those with 4 or more years of education, non-white, in the second tertile of family income and those living alone. Despite a reduction in the prevalence of one or more self-reported chronic conditions, the prevalence of hypertension increased from 44.1 % (1998), to 49.0 % (2003) and then to 53.4 % (2008) (*p* < 0.001). A similar pattern was observed for the prevalence of diabetes from 10.3 % (1998), to 13.0 % (2003) and then to 16 % (2008) (*p* < 0.001). In addition, it was observed the increase of the number of physician visits and the reduction of hospitalizations over the ten year period.

**Table 1:** Socio-demographic characteristics, health status and use of health services among Brazilian older adults (PNAD - 1998, 2003 and 2008)

Variable	Year			
	1998 (%)	2003 (%)	2008 (%)	*p*-value
Gender: Female	55.6	56.0	56.1	0.274
Age (years)				
60-69	57.3	55.5	55.4	<0.001
70-79	30.7	32.1	31.0	
80+	12.0	12.3	13.5	
Schooling (years)				
Illiterate	41.5	37.2	32.8	<0.001
1 to 3	22.2	21.2	19.7	
4 or more	36.7	41.6	47.5	
Self-declared skin colour: white	61.0	59.2	55.6	<0.001
Family Income in Brazilian minimum wages (tertile)				
First	42.1	45.3	43.5	<0.001
Second	19.6	21.5	24.2	
Third	38.8	33.1	32.1	
Household Composition: live alone	11.9	13.0	14.0	<0.001
Diabetes	10.3	13.0	16.0	<0.001
Hypertension	44.1	49.0	53.4	<0.001
One or more self-reported chronic disease^a^	69.2	60.3	58.2	<0.001
Number of physician visits in the last 12 months				
None	27.8	21.8	18.7	<0.001
1-3	39.1	38.3	39.4	
4 or more	33.1	39.8	41.9	
One or more hospitalizations in the last 12 months	13.7	12.7	12.3	<0.001

Note: *p*-values were derived from chi-square tests. ^a^Chronic disease: back problems, arthritis, cancer, diabetes, bronchitis or asthma, hypertension, heart disease, chronic kidney disease, depression, tuberculosis, tendinitis and cirrhosis

The physical mobility indicators measured in this study are described in Table 2. There was a significant association between year of survey and all the mobility tasks considered. The Physical Mobility Index showed an increase in the group without difficult and a decrease of the proportion in the moderate difficult group, over the period considered.

**Table 2:** Physical mobility indicators among Brazilian older adults (PNAD - 1998, 2003 and 2008)

Mobility tasks/level of difficulty	Year			
	1998 (%)	2003 (%)	2008 (%)	*p*-value
Running, lifting heavy objects, playing sports or doing heavy work				
None	30.6	34.8	34.3	<0.001
Little	22.8	21.0	21.4	
Great	25.1	23.6	23.0	
Can not	21.5	20.6	21.2	
Pushing a table or doing a home repair				
None	55.4	58.5	52.7	<0.001
Little	22.7	20.0	21.8	
Great	11.0	10.5	12.9	
Can not	11.0	11.0	12.6	
Going up a steep hill or stairs				
None	40.4	45.5	45.1	<0.001
Little	25.6	22.6	23.4	
Great	21.5	19.2	18.9	
Can not	12.5	12.6	12.5	
Stooping or Kneeling				
None	44.7	48.7	45.8	<0.001
Little	25.7	22.6	24.1	
Great	18.7	17.3	18.7	
Can not	11.0	11.3	11.4	
Walking about 100 m				
None	74.8	77.3	72.0	<0.001
Little	12.4	10.5	14.3	
Great	4.9	4.6	5.6	
Can not	7.8	7.6	8.1	
Physical Mobility Index				
No difficulty	24.6	28.0	28.5	<0.001
Moderate difficulty	38.7	37.4	35.2	
Greater difficulty	36.7	34.6	36.3	

Note: *p*-values were derived from chi-square tests

The results of the multinomial logistic regression examining the association between the index of physical mobility, hypertension and diabetes are given in Table 3. Considering the adjusted model, respondents in the moderate difficulty group showed a discrete reduction in the strength of the association between both chronic diseases and physical mobility, but this trend was not significant (*p* > 0.05 for this category). On the other hand, the group with great difficulty to perform mobility activities showed a reduction in the strength of the association from 1998 to 2003 (*p* < 0.05), but this trend was not sustained in the last five years.

**Table 3:** Results of the multinomial logistic regression analyses examining the association between physical mobility, hypertension and diabetes in a representative sample of Brazilian older adults (PNAD - 1998, 2003 and 2008)

Disease/Mobility	1998		2003		2008	
	OR (95 % CI)		OR (95 % CI)		OR (95 % CI)	
	Model 1	Model 2	Model 1	Model 2	Model 1	Model 2
Hypertension						
No difficulty	1.00	1.00	1.00	1.00	1.00	1.00
Moderate difficulty	2.15 (1.99-2.34)	1.51 (1.39-1.65)	2.07 (1.93-2.21)	1.44 (1.34-1.56)	1.89 (1.77-2.01)	1.37 (1.28-1.46)
Great difficulty	3.89 (3.60-4.21)	2.01 (1.83-2.21)	3.45 (3.22-3.70)	1.75 (1.61-1.90)	3.28 (3.08-3.49)	1.76 (1.64-1.89)
Diabetes						
No difficulty	1.00	1.00	1.00	1.00	1.00	1.00
Moderate difficulty	1.90 (1.63-2.21)	1.44 (1.23-1.69)	1.67 (1.50-1.88)	1.19 (1.05-1.35)	1.57 (1.43-1.72)	1.21 (1.10-1.33)
Great difficulty	3.43 (2.97-3.97)	2.07 (1.75-2.44)	2.88 (2.59-3.20)	1.56 (1.37-1.77)	2.82 (2.58-3.08)	1.80 (1.62-2.00)

OR (95 % CI): odds ratio (confidence interval at 95 %). Model 1 adjusted for time-disease interaction term. Model 2 adjusted for time-disease interaction term, sex, age, education, race, family components, income, number of diseases (except hypertension for hypertension model and except diabetes for diabetes model), number of physician visits, hospitalization

## Discussion

The main findings from the present study showed a significant association between physical mobility and two chronic conditions (hypertension and diabetes) in three nationally representative sample of Brazilian older adults. In addition, despite an increase in the prevalence of hypertension and diabetes from 1998 to 2008, the magnitude of the association between these chronic conditions and physical mobility did not change among those in the moderate difficulty group. However, among those with great difficulty, there was a decrease in the magnitude of these associations from 1998 to 2003, but our findings showed no change in the magnitude of the association for hypertension and a small increase in the strength of the association for diabetes, between 2003 and 2008.

The increase in prevalence of diabetes and hypertension among older adults in Brazil is similar to those observed in the USA [27] and Holland [22] in the same age group. Among American older adults data came from two national surveys namely the Health and Retirement Study (HRS) and the Asset and Health Dynamics (AHEAD) from years 1998, 2004 and 2008, showing an increase in the prevalence of these two chronic conditions [27]. Similar findings were reported from a prospective Dutch study between 1992 and 2009 [22]. This increase observed in Brazil and other countries could be partly attributed to the implementation of new diagnostic criteria, improvements in treatments and more access to health care, leading to a better management of these two chronic diseases and, consequently, less health-related complications such as disability [27].

Overall, more than 20 % of the respondents did not report any difficulty to perform any of the five mobility tasks according to the physical mobility index (PMI) used in this study. The literature on changes over time in the prevalence of indicators of mobility shows inconsistencies. In the USA, a reduction was observed in the physical mobility decline among older adults (aged 65 and over) between 1982 and 1994 [28] and also among those aged 70 and over between 1982 and 2005 [29]. On the other hand, data from the American people aged 65 and over between 2000 and 2005 showed an increase in the prevalence of physical mobility difficulties [30]. Similar findings were reported from a study conducted in Singapore among residents aged 55 and over (1995–2005) [1]. Such differences in the prevalence changes of indicators of physical mobility over time could be due to differences in sample designs, measures used and analytical approaches as well as changes in the health conditions over time [1]. These highlight the difficulty in establishing a general pattern of change in physical mobility among older adults from different populations.

The findings showed a significant association between hypertension and worsening physical mobility. Studies that assessed mobility using objective physical measurements also found an association with hypertension, which corroborate our findings [9, 10]. An American prospective study between 1989 and 2007 conducted with 643 older adults showed that higher systolic blood pressure was associated with a rapid decline in walking speed [9]. Data from a Swedish study conducted between 2001 and 2004 in 2725 older adults aged 60 and over also showed an association between cardiovascular diseases (including hypertension) and limitations in mobility, defined as walking speeding. They also reported that those respondents with two or more cardiovascular diseases were three times more likely to have mobility difficulties [10]. The hypertension has silent symptoms but when uncontrolled, it can lead to the evident symptom, which increases the probability of a decline in physical mobility [31]. The possible explanation for this association is through its effect on white matter hyperintensities in the brain, cerebrovascular function, overall lean muscle mass, inflammation or changes in the renin angiotensin system [31]. White matter hyperintensities are closely related to hypertension and have been connected with mobility impairment [7]. This could be a potential explanation for the association between hypertension and decline in physical mobility.

Similarly in relation to hypertension, there was a significant association between physical mobility status and diabetes in the present study. A prospective study, conducted in Spain between 2000 and 2007 in older adults aged 65 and over, showed a significant association between diabetes and mobility difficulties assessed by self-reported difficulty to walk for an hour without resting and climbing ten steps [8]. Similar findings were observed in a Mexican population of older adults living in the USA followed up for seven years (1993–2001). Mobility was measured by questions about difficulties to climb steps and walking half a mile [32]. Studies that assessed mobility using objective physical tests also reported a significant association with diabetes [12, 33] illustrating the importance of diabetes in the decline of physical mobility. In a cross-sectional study conducted among Italian older adults enrolled in the inCHIANTI (Invecchiare in Chianti, aging in the Chianti area) the mobility was assessed by 4 m and 400 m walking speed, and the diabetics participants were significantly slower when compared to non-diabetics [33]. Another cross-sectional study, in southern Netherlands, the authors evaluated the mobility by a 6 min walk test and timed up and go test, observed that diabetics had a decrease in mobility compared to the non-diabetics [12]. Therefore, it is crucial to detect diabetes in its early stage in order to avoid health-related complications that will lead to a decline in physical functioning [34].

Overall, our findings showed a decrease in the magnitude of changes in the associations among those with great difficulty, from 1998 to 2003, which was not observed between 2003 and 2008. A study from the USA reported similar findings to ours namely a reduction in the association between hypertension and the decline in mobility in the years 1998, 2004 and 2008. In addition, a reduction was found in the strength of the association between diabetes and mobility decline from 1998 to 2004 and an increase in the strength of the association in 2008. These findings corroborate ours and highlight the fact that chronic diseases are highly associated with disability. One should note the recent increase of the association between diabetes and difficulties in physical mobility [23]. Thus, studies looking into changes in the strength of the association between chronic conditions and physical functioning over time are essential to provide information about the impact of such diseases on levels of physical mobility among older adults and, consequently, helping health services to prioritize the best interventions [23, 32].

In order to minimise the health-related complications caused by diabetes and hypertension, the Brazilian Ministry of Health implemented a series of public health interventions at a primary care level in 2002. One of them is the free and continuous distribution of medication for the treatment of diabetes and hypertension as well as the monitoring of clinical aspects of patients with such conditions using the free Brazilian national health service (SUS) [35, 36]. However, there is a need to further increase such intervention in order to reduce the impact of both diabetes and hypertension on physical mobility decline found in the present study. Another concern is the reduction in the quality of life of older adults around the world as a result of the increase in the magnitude of the association between diabetes and difficulties in physical mobility and the increase in the prevalence of diabetes [23, 32].

Our study has some limitations. Its cross-sectional nature does not allow establishing temporal causation, although the literature shows that both diabetes and hypertension are associated with a decline in physical mobility [7, 37]. Chronic disease was based on self-report of physician diagnosis, though previous work has demonstrated the validity of this measure especially among older adults [32, 38, 39]. The medication compliance variable is not available from the primary dataset. This variable is important for assessing association between chronic disease and mobility, because the use of medication can help to attenuate, prevent or retard disability [31]. Finally, in 1998, the self-reported diagnosed question was different from the one used in years 2003 and 2008. This change should however is not likely to alter the estimates of prevalence of the chronic conditions as discussed in a previous study [40]. On the other hand, it is important to highlight that the sample used in this analysis is nationally representative of the Brazilian older adult population from all geographic regions. PNAD collects important data to assess the potential impacts of policies implemented by the Brazilian government to improve both the health and socioeconomic conditions of the Brazilian population [41]. Another strength of this study is the use of a score that summarises five physical mobility indicators based on the Item Response Theory (IRT). Very few studies have used the IRT to investigate physical functioning [42–44]. This approach generates a scale that allows differentiation of respondents according to their different levels of physical mobility taking into account various questions that measure this domain [44].

## Conclusions

In summary, this study revealed that among Brazilian older adults there was an increase in the prevalence of both hypertension and diabetes from 1998 to 2008. Our findings also showed that there was a trend of reduction in the strength of the association between these two chronic conditions and great difficulty in physical mobility in the first five years, indicating a relative improvement in public health policies to better manage these chronic conditions in Brazil, but this trend was not observed between 2003 and 2008. Nevertheless, this study was able to demonstrate that diabetes, hypertension and physical mobility are important issues for older adults in Brazil and that their relationship is an area that needs to be researched further in order to improve our understanding of the mechanisms through which they impact upon the individual and society. This in turn would enable the development of appropriate policies and target interventions effectively to prevent declines in physical mobility. The fact that both chronic diseases studied have been shown to affect a high proportion of individuals in later life is relevant to public policy.

## Abbreviations

ADL: Activities of daily living
IADL: Instrumental activities of daily living
PNAD: National Household Sample Survey
IBGE: Brazilian Institute of Geography and Statistics
PMI: Physical mobility index
TRI: Item Response Theory
HRS: Health and Retirement Study
AHEAD: Asset and Health Dynamics
